# Effect of vaccination against Covid-19 one year after its introduction in Brazil

**DOI:** 10.1186/s40794-022-00183-5

**Published:** 2022-11-18

**Authors:** Jadher Percio, Cibelle Mendes Cabral, Francieli Fontana Sutile Tardetti Fantinato, Dalva Maria de Assis, Lely Stella Guzmán-Barrera, Wildo Navegantes de Araújo

**Affiliations:** 1 University of Brasília, UNB, Darcy Ribeiro University Campus, Gleba A, North Sector, Via L3 North, CEP: 70.723-040, Brasília/DF, Brazil; 2 Department of Health Surveillance, Ministry of Health, SRTV 702, Via W5 North, CEP: 70.723-040, Brasília/DF, Brazil; 3 Pan-American Organization / World Health Organization (PAHO/WHO) in Brazil, Lot 19 - Avenida das Nações, SEN - Asa Norte, Brasília/DF, 70312-970 Brazil

**Keywords:** Covid-19. Pandemic, Vaccination programs, COVID-19 vaccines, Vaccination coverage, Time series analysis, Brazil

## Abstract

**Background:**

Worldwide, several efforts have been made to develop, distribute and administer safe and effective vaccines to reduce morbidity and mortality and control the Covid-19 pandemic. This study aimed to analyze the effect of vaccination against Covid-19, one year after its introduction in Brazil.

**Methods:**

An ecological study that analyzed the general effect of vaccination against Covid-19 on disease morbidity and mortality indicators among the Brazilian population aged 18 years or older per epidemiological week (EW), comparing the pre and postvaccination period. Morbidity and mortality indicators were calculated from secondary databases (hospitalization rate, severity, case fatality rate and mortality) and vaccination coverage by age groups (18 to 59 years and 60 years or older). Morbimortality trends were estimated using the JoinPoint model and their association with vaccine coverage using the Poisson model.

**Results:**

The average weekly percentage change (AWPC) of morbidity and mortality indicators reduced after the introduction of Covid-19 vaccination: hospitalization rate (from 15.3% to -6.0%), severity (from 0.4% to -0.2%), case fatality rate (from 0.3% to -0.2%) and mortality (from 20.5% to -4.3%). The following indicators were inversely associated with the increase in vaccine coverage against Covid-19: hospitalization (IRR: 0.974), mortality (IRR: 0.975) and lethality for people aged 60 years or older (IRR: 0.997).

**Conclusions:**

In spite of the three epidemic waves and the circulation of variants of concern, the general effect of vaccination against Covid-19 in reducing the trend of morbidity and mortality from the disease in Brazil was demonstrated. These findings contribute to a better understanding of the mass vaccination program against Covid-19 and may inform future public health policies.

## Background

The Coronavirus Disease 2019 (Covid-19), whose etiological agent is the Severe Acute Respiratory Syndrome Coronavirus 2 (SARS-CoV-2), was responsible for the biggest public health emergency in recent centuries – the Covid-19 pandemic [1].

Since the beginning of the Covid-19 pandemic in 2020, several efforts have been made worldwide for the accelerated development of safe and effective vaccines initially aimed at reducing hospitalizations and deaths from the disease [2].

Vaccines represent one of the most cost-effective technologies for disease prevention and control, contributing greatly to strengthening global health and consequently, restoring the economy of countries that were impacted, directly and indirectly, by the Covid-19 pandemic [3].

The World Health Organization (WHO), in December 2020, approved the first COVID-19 vaccines for emergency use, which are now massively purchased and administered by countries, depending on the global availability of vaccines and supplies [4].

Each country was responsible for preparing its National Operational Plan (NOP) for vaccination against Covid-19, which was mainly guided by the WHO recommendations and generally implemented by the National Immunization Programs (NIP) [5, 6].

The effect of vaccines can be measured both at the individual level, considering the result observed in susceptible populations, infected individuals and on disease progression; and at the population level, depending on vaccination coverage, the distribution of vaccines and the interaction between different population groups (vaccinated and unvaccinated, for example) [7].

In this respect, several studies have been published about the effect of COVID-19 vaccines on individuals. On the other hand, the evidence available on the effect of vaccination against Covid-19 at the population level is scarce [8].

Mathematical models have estimated that to achieve herd immunity against SARS-CoV-2, approximately 65–70% of the population should be immunized against Covid-19 [9].

In Brazil, in 2020, the seroprevalence of antibodies against SARS-CoV-2 was considered low, ranging from 3 to 15%, totaling approximately 212 million people infected before the start of vaccination against Covid-19, which in this country, began on January 17, 2021 [10, 11].

Four months after the start of vaccination against Covid-19 in Brazil, about 95% of Brazilians aged 80 or older had already received at least the first dose of the vaccine. During this period, there was a reduction in the proportionality of deaths from Covid-19 among people aged 80 years or older which reduced from 25 to 30% at the beginning of vaccination, to 13%, after four months of vaccination against Covid-19 in the country [12].

However, after three years of the pandemic, Brazil was responsible for at least 10 of every 100 deaths from Covid-19 recorded worldwide by the WHO [13]. Therefore, this study aimed to estimate the effect of vaccination against Covid-19 in Brazil, after more than a year of the introduction and administration of COVID-19 vaccines throughout the country.

## Methods

### Study design

This is an ecological study that analyzed the temporal trend of morbidity and mortality of Covid-19 in Brazil, comparing two periods: before (pre) and after (post) the introduction of vaccination against Covid-19. The effect of vaccination on vaccinated and unvaccinated people was analyzed in combination with its outcome at the population level, that is, the overall effect of the NOP of vaccination against Covid-19 in the country [7].

### Context

In Brazil, the NOP for vaccination against Covid-19 was guided by the Ministry of Health and executed by the Municipalities with the support of the States, through the Unified Health System (SUS, in Portuguese). The SUS guarantees access to health services of the Brazilian population at all levels of health care and health surveillance, in a cost free, universal and equitable manner [14].

In Brazil, four different COVID-19 vaccines were introduced that were developed on three different platforms: i) non-replicating viral vector (AstraZeneca/Oxford University and Janssen Pharmaceutical), ii) inactivated virus (Sinopharm) and iii) messenger RNA- -mRNA (Pfizer/ BioNTech). These vaccines have particular indications of vaccination schedule (single dose, two doses and additional or booster doses) and target population. Additionally, they have different efficacy, effectiveness and safety results [15–18].

Initially, elderly people (60 years or older), with health conditions that increase the risk for severe illness, health professionals and other groups of greater vulnerability to the disease were contemplated for vaccination against Covid-19 in Brazil. Later, in May 2021, the population aged 18 to 59 began to be vaccinated according to the guidelines of the Brazilian NOP [19]. Subsequently, in June 2021 the vaccination of adolescents (12 to 17 years old) was started and in December 2021, children aged 5 to 11 years were also included for vaccination against Covid-19 in the country [11].

The variants of concern (VOCs) or of interest (VICs) of the SARS-CoV-2 virus that circulated in Brazil were as follows: Gama (former P.1, identified in Dec/2020), Delta (former B.1.617. 2, identified in Jun/2021) and Omicron (B.1.1.529, identified in Dec/2021) [20].

### Sources and use of data

#### Influenza Epidemiological Surveillance Information System (SIVEP-Gripe, in Portuguese)

This is the official system for the registration of hospitalized cases and deaths of Severe Acute Respiratory Syndrome (SARS) in Brazil, whose database is made available without nominal identification of cases (anonymized data) through the link: https://opendatasus.saude.gov.br/organization/ministry-of-health.

The databases used in the study (SARS-2020 and SARS-2021) were obtained on August 8, 2021. Data such as date of onset of symptoms, age (18 to 59 years / 60 years or older), admission in the Intensive Care Unit (ICU), use of invasive ventilatory support, final disease classification (Covid-19), confirmation criteria (laboratory), case evolution (death) and date of evolution were used for analysis.

#### National Health Data Network (RNDS, in Portuguese)

It integrates different databases on records of doses of COVID-19 vaccines administered in Brazil, including the National Immunization Program Information System (SI-PNI, in Portuguese), the Primary Health Care Information System (e-SUS APS, in Portuguese) among others.

The database was obtained for analysis on August 8, 2021, through the link: https://localizasus.saude.gov.br/. Data such as date of vaccination, age (18 to 59 years / 60 years or older) and dose type (dose 1, dose 2, single dose or booster dose) were used for analysis.

#### Brazilian Institute of Geography and Statistics (IBGE, in Portuguese)

Population estimates were obtained by age groups (18 to 59 years / 60 years or older) for the analyzed period. Data are available through the link: https://www.ibge.gov.br/.

### Selection criteria

Hospitalized cases of Covid-19, confirmed by laboratory criteria, reported in SIVEP-Gripe, in the following age groups were selected: i) 18 to 59 years, and ii) 60 years or older; and had the onset of symptoms or died between February 16, 2020, and April 2, 2022.

The choice of this period considered the introduction of the SARS-CoV-2 virus in Brazil, in 2020 and the first year after the start of vaccination against Covid-19counted from January 18, 2021 (EW 3/2021) with an addition of three more months to include the period of dissemination of the Omicron variant in the country.

### Variables

The following variables were processed and analyzed in the study:

- Hospitalization rate per 1,000,000 inhabitants: the numerator used was the number of hospitalized cases per EW of symptom onset and the denominator used was the number of people residing in the country per age group.
- Severity (%): the numerator used was the number of cases admitted to the ICU and/or who received invasive ventilatory support, and the denominator applied was the number of hospitalized cases, disaggregated by age group and EW of symptoms onset.
- Case fatality rate (%): the numerator used was the number of deaths and the denominator used was the number of hospitalized cases, separated by age group and EW of symptoms onset.
- Mortality per 1,000,000 inhabitants: the numerator used was the number of deaths per EW on the date of evolution and the denominator used was the number of people residing in the country per age group.
- Vaccination coverage (%): the numerator used was the number of people per age group with a complete vaccination schedule (two doses or a single dose) of any COVID-19 vaccine established in the NOP and the denominator used was the number of people residing in the country per age group and year.

### Data analysis

#### JoinPoint regression model

Trends and temporal changes were estimated for all indicators using the JoinPoint regression model. Inflection points (joinpoints) in temporal trends and regression coefficients were estimated while the ideal number of joinpoints was selected through a permutation test, estimated by the traditional Bayesian Information Criteria (BIC3) method, considering a level of statistical significance of < 0.05 [21].

The EWs were attributed as an independent variable and indicators of morbidity and mortality of Covid-19 as dependent variables. In summary, time trends were converted into Weekly Percentage Changes (WPC); for example, the temporal change of an indicator of morbidity and mortality from Covid-19, estimated from one joinpoint to the next, can be estimated as a percentage of weekly increase, when positive or weekly decrease, when negative.

To allow a detailed comparison, a weighted average of the combined WPC, the Average Weekly Percentage Change (AWPC), was calculated for the pre-vaccination (EW 8/2020 to EW 2/2021) and post-vaccination periods (EW 3/2021 to EW 13/2022). The 95% confidence intervals (95% CI) of the AWPC were also calculated for the analyzed periods.

#### Poisson’s regression model

Simple Poisson regression models were used to analyze the association between vaccination coverage (dependent variable) and Covid-19 morbidity and mortality indicators (independent variables). Incidence-rate ratios (IRR) and their respective 95% CI were calculated, considering a significance level, < 0.05. The model fit was evaluated by the *pseudo R2* statistical method, which measured the reduction in deviation due to the explanatory variable.

### Ethical aspects

The databases obtained for analysis in this study are publicly accessible and do not have variables that could identify the population studied. In this regard the study was exempted from ethical review by a Human Research Ethics Committee according to current legislation in Brazil.

## Results

During the study period, there were 2,853,679 reports of SARS hospitalizations among people over 18 years of age, of which 1,742,473 (61.1%) were laboratory confirmed for Covid-19, including 624,747 (35.9%) cases considered severe and 563,821 (32.4%) deaths from the disease.

### Analysis of time trends: 60 years and older

**Fig. 1:**
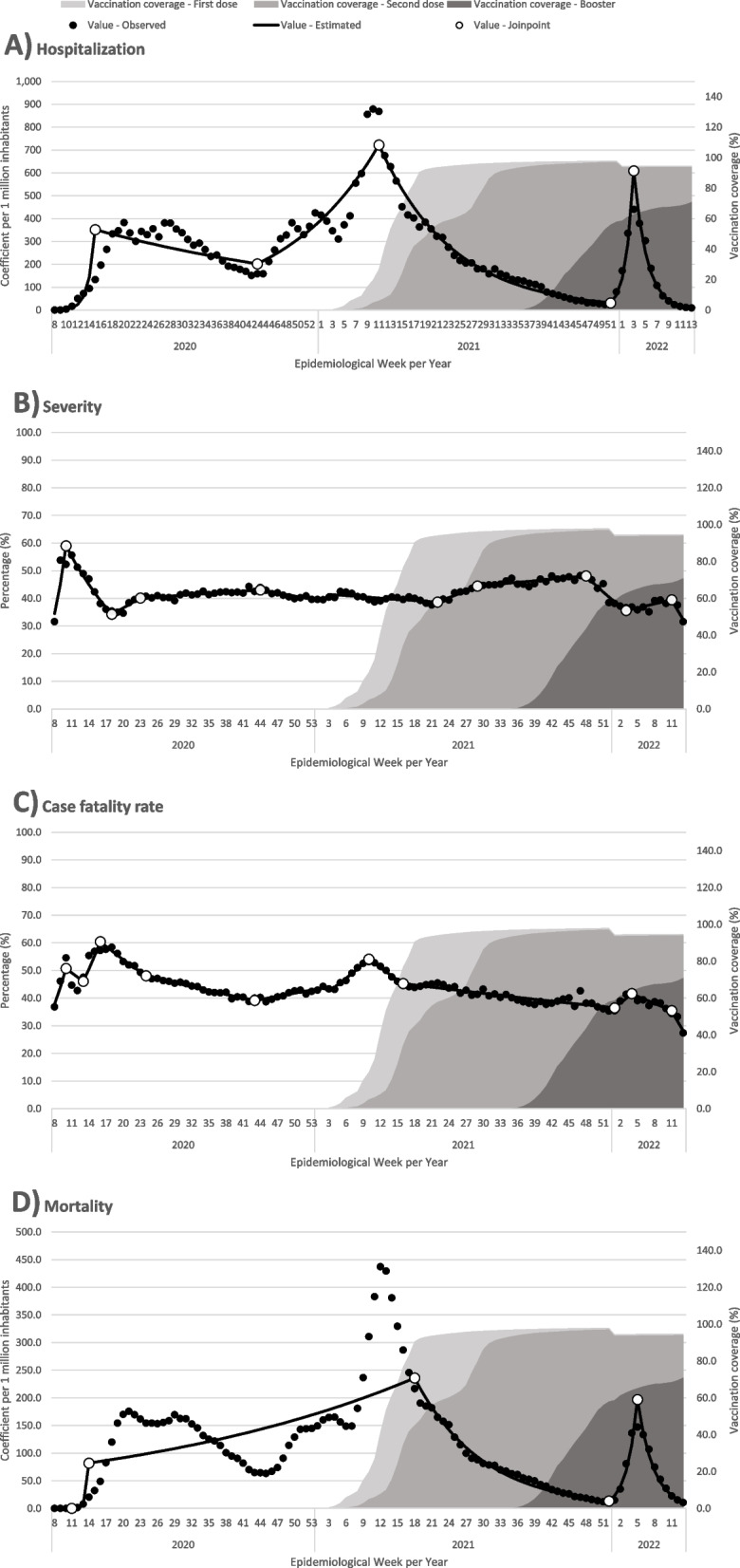
Temporal trends in Covid-19 morbidity and mortality indicators and Covid-19 vaccine coverage for people aged 60 or older. Brazil, 2020–22

The time series of morbidity and mortality and vaccination against Covid-19 for people aged 60 years or older can be seen in Fig. 1. The estimated WPC for each time interval between two joinpoints can be seen in detail in Appendix Table 3.

The distribution of hospitalizations and deaths in this population group showed the occurrence of three epidemic waves in the analyzed period. On the other hand, severity and case fatality rate showed less temporal variability.

The first wave had its peak of hospitalizations in EW 20/2020 (382.92 hospitalized cases per 1 million inhabitants) and lasted until EW 43/2020 when the second wave began, reaching its maximum point in EW 11/ 2021 (868.91 hospitalized cases per 1 million inhabitants). The third wave started in EW 51/2021 and reached its peak in EW 3/2022 (609.2 hospitalized cases per 1 million inhabitants).

Regarding the temporal trend of mortality, the first peak was observed in EW 21/2020 (175.58 deaths per 1 million inhabitants), the second was reached in EW 13/2021 (429.39 deaths per 1 million inhabitants) and the third took place in EW 5/2022 (196.69 deaths per 1 million inhabitants).

After the introduction of vaccination against Covid-19, in January 2021 (EW 3), until March 2022 (EW 13), the cumulative vaccination coverage for people aged 60 years or older was 94.7% for the first dose, 94.0% for the second dose and 70.9% for the booster dose.

## Analysis of time trends: 18 to 59 years

**Fig. 2:**
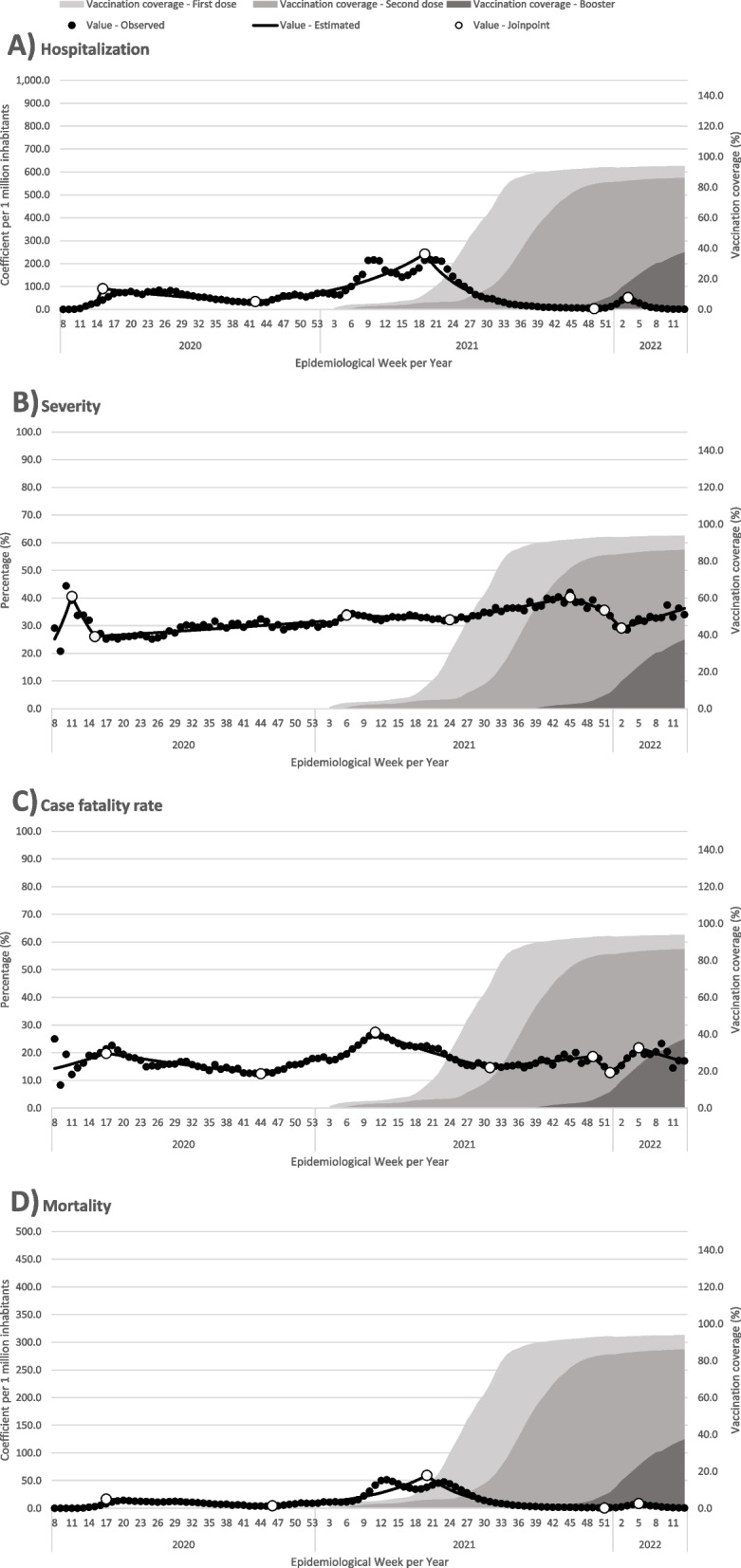
Temporal trends in Covid-19 morbidity and mortality indicators and Covid-19 vaccine coverage for people aged between 18 and 59. Brazil, 2020–22

In addition, the time series of morbidity and mortality and vaccination against Covid-19 for people aged 18 to 59 years can be observed in Fig. 2. On the other hand, the estimated WPC for this population group, for each time interval between two joinpoints, can be verified in detail in Appendix Table 4.

Unlike that observed with older people, the distribution of hospitalizations and deaths in this population group was mild, and only one epidemic wave was detectable in the analyzed period. The peak of hospitalizations occurred in EW 19/2021 (242.38 hospitalized cases per 1 million inhabitants), therefore, the peak in mortality occurred in EW 20/2021 (59.63 deaths per 1 million inhabitants). Severity and case fatality rate also showed less temporal variability in this group.

Accumulated vaccination coverage for people aged 18 to 59 at the end of the study period was 93.9% for the first dose, 86.1% for the second dose and 37.2% for the booster dose.

## Analysis of the effect of vaccination against Covid-19 on temporal trends in morbidity and mortality

The regression analysis using the Joinpoint model identified that, in both groups analyzed (Table 1), there was a weekly trend of increased morbidity and mortality in the pre-vaccination period (*p*-value < 0.05) and a reduction in the values observed in the post -vaccination period (*p*-value < 0.05); except for the case fatality rate in people aged 18 to 59 years in the last analyzed period (*p*-value > 0.05).

**Table 1:** Temporal trends of Covid-19 morbidity and mortality indicators in the pre- and post-vaccination periods per age groups and in total. Brazil, 2020–22

Indicators	Population (age in years)	Pre-vaccination				Post-vaccination			
		AWPC	95% CI		*P*-Value	AWPC	95% CI		*P*-Value
			Low	High			Low	High	
Hospitalization	18 to 59	15.3	12.4	18.3	< 0.001	-6.6	-7.8	-5.4	< 0.001
	60 or older	15.8	13.1	18.5	< 0.001	-5.6	-6.6	-4.7	< 0.001
Severity^a^	18 to 59	0.5	-0.8	1.8	< 0.001	0.3	-0.2	0.8	< 0.001
	60 or older	0.3	-0.1	0.6	< 0.001	-0.4	-0.7	-0.1	< 0.001
Case fatality rate	18 to 59	0.6	-0.5	1.7	< 0.001	0.0	-1.1	1.2	1.000
	60 or older	0.3	-0.1	0.8	< 0.001	-0.7	-1.2	-0.1	< 0.001
Mortality	18 to 59	18.5	15.9	21.2	< 0.001	-4.0	-5.3	-2.8	< 0.001
	60 or older	21.5	17.8	25.2	< 0.001	-4.3	-5.4	-3.1	< 0.001

^a^Covid-19 cases admitted to the ICU and/or who received invasive ventilatory support

Regarding the regression analysis using the Poisson model, it was demonstrated that, for all groups analyzed, vaccination coverage was inversely associated with the hospitalization rate (*p*-value < 0.05), with the case fatality rate (*p*-value < 0.05) and with mortality (*p*-value < 0.05) associated with Covid-19; except for severity (*p*-value > 0.05). The models that were statistically significant (*p*-value < 0.05) explained between 1.6% and 72.4% of the observed variance of the outcome (Table 2).

**Table 2:** Association of vaccine coverage with Covid-19 morbidity and mortality indicators per age groups and in total. Brazil, 2020–22

Indicators	Population (age in years)	IRR	95% IC		*P*-value	Pseudo R2
			Low	High		
Hospitalization	18 to 59	0.965	0.963	0.966	< 0.001	72.4%
	60 or older	0.982	0.982	0.983	< 0.001	63.0%
Severity^a^	18 to 59	1.001	1.000	1.002	0.238	0.4%
	60 or older	1.001	1.000	1.002	0.205	0.4%
Case fatality rate	18 to 59	0.998	0.997	1.000	0.019	1.6%
	60 or older	0.997	0.996	0.998	< 0.001	6.5%
Mortality	18 to 59	0.965	0.962	0.969	< 0.001	62.0%
	60 or older	0.983	0.983	0.984	< 0.001	58.7%

^a^Covid-19 cases admitted to the ICU and/or who received invasive ventilatory support

## Discussion

The present study estimated the population effect of vaccination against Covid-19 in Brazil, more than a year of implementation of the NOP, analyzing the association of vaccination coverage with the temporal trend of morbidity and mortality indicators. Two population groups (people aged 18 to 59 and aged 60 years or older), were analyzed by comparing temporal trends in the pre- and post-vaccination periods.

In summary, our findings indicate that vaccination against Covid-19 had an influence on the reduction of hospitalizations and deaths from the disease in Brazil. The hospitalization rate and mortality per 1 million inhabitants had a strong inverse association with vaccination coverage. This association was statistically significant in both groups analyzed. In contrast, vaccination had less influence on severity and case fatality rate.

The most intense epidemic wave of Covid-19 was observed in the age group of 60 years or older and occurred during the predominance of the Gamma variant, eight weeks after the start of vaccination against Covid-19 in the country (EW 11/2021). In that period, vaccination coverage for the first dose was 26.8% and for the second dose it was 6.0%, and there was still no official recommendation for the booster dose.

Therefore, the third epidemic wave, observed in the same group of people, occurred exactly one year after the start of vaccination against Covid-19 (EW 3/2022), when vaccination coverage for people aged 60 years or older was 94, 6.0% for the first dose, 93.7% for the second dose and 62.8% for the booster dose. At that time, the circulation of the Omicron variant predominated in the country.

However, the regression models in this study demonstrated a statistically significant effect in reducing the risk of hospitalizations and deaths from Covid-19 with the increase in vaccine coverage in Brazil. In the case of people aged 60 years or older, for example, with each weekly percentage increase in vaccination coverage, the hospitalization rate was reduced by 0.02% (IRR: 0.982), the case fatality rate at 0.01% (IRR: 0.997) and the mortality rate at 0.02% (IRR: 0.983).

A study that evaluated the initial impact of vaccination on the Covid-19 pandemic in the United States of America (USA) showed that vaccination also significantly slowed the increase of cases and hospitalizations for the disease. It was demonstrated that an additional increase of one person vaccinated per 100 inhabitants (with two doses) reduced by 1.1% the weekly incremental rates of cases and hospitalizations Based on these estimates, vaccination reduced the number of new cases by 4.4 million and by 0.12 million cases hospitalizations in the US initially [22].

Another study showed that in six countries (Israel, United Arab Emirates, Chile, Hungary, Qatar and Serbia), where at least 50% of their population had been vaccinated, peaks of Covid-19 occurred after the start of vaccination and before obtaining herd immunity. However, they concluded that vaccination against Covid-19 contributes to the reduction of cases of the disease, ranging from 1.46 to 50.91%, and their models explained the variability of outcomes from 57.2% to 89.9% [23].

This condition may have been aggravated by the circulation of highly adapted and transmissible variants, such as Delta and Omicron, and by the low coverage of the booster dose whose objective was to boost the immunity of those who had been earlier vaccinated against Covid-19, especially in older people and those with comorbidities.

VOCs or VICs invariably present greater virulence or transmission capacity, and may even escape the immunity already acquired (via vaccine or natural infection), contributing to decrease in the effectiveness of COVID-19 vaccines [24–26].

In the UK, a study demonstrated that the effectiveness of COVID-19 vaccines for the Delta variant compared to the Alpha variant was reduced by 37% for the first dose and 6 to 10% for the second dose [27]. Similar results were found in relation to the Omicron variant [25].

Our study showed that vaccination coverage for booster doses was incipient and had slow advancement in Brazil. Although the supply and availability of vaccines has progressively increased, vaccine hesitancy and the feeling of security by the population brought about by the rapid decrease in cases may be related to low adherence to booster doses [11, 28].

With decrease in the effectiveness of vaccines against new variants, the vaccination coverage needed to achieve herd immunity increases [23]. A study conducted in the USA showed, for example, that for a vaccine with an effectiveness of 80%, at least 82% of the population should be immunized to achieve herd immunity to the point of reducing deaths from Covid-19 [9].

The efficacy of the vaccines used in Brazil, before the circulation of the Delta and Omicron variants, ranged from 54 to 95% to prevent infection with SARS-CoV-2 and from 67 to 76% to prevent moderate to critical cases of Covid-19 [15–18]. To achieve the much-desired herd immunity in Brazil, vaccination coverage must be high and maintained in all population groups.

To the best of our knowledge, this is the first published study that used this method to analyze the population effect of vaccination against Covid-19. In this regard, this study contributes to findings of national and international interest, especially when considering the importance of a country of continental proportions like Brazil.

However, it should be noted that the method applied in this study has already been used by other researchers to analyze the population effect of vaccination on the control and prevention of other vaccine-preventable diseases, such as pneumococcal disease and chickenpox, for example [29, 30].

The interpretation of the results of this study must consider some limitations imposed, mainly, by the use of administrative data that may have underestimated the analyzed indicators, especially vaccination coverage, which depends on timely registration of disease cases and also the vaccinated cases in their respective information systems.

It is worth highlighting that individual data were not analyzed while comparing the rates among vaccinated people in relation to those who were not vaccinated, stratified by the number of doses received. Thus, it is important to note that the statistical association observed in this study does not reflect the individual effect of vaccination against Covid-19.

Finally, it should be considered that other factors, such as non-pharmacological measures – use of masks, social distancing, mass testing, among other interventions – may also have played an important role in controlling the pandemic in Brazil.

## Conclusion

The findings of this study demonstrate that based on epidemiological surveillance data, the vaccination against Covid-19 had an important effect in controlling the epidemic in Brazil. This study suggests that, with the circulation of new variants of SARS-CoV-2 and the consequent decrease in the effectiveness of vaccines, new waves of Covid-19 may occur even with high vaccine coverage.

The fight against the pandemic continues to be an important challenge; therefore, it is necessary to consider making every effort to increase vaccine coverage, especially in relation to booster doses, and to invest heavily in the development of new, safe and effective vaccines to combat the new variants.

## Data Availability

Datasets used and/or analyzed during the current study are available from the corresponding author upon reasonable request. Furthermore, the original databases are publicly accessible and can be found through the links mentioned in the methods section.

## Appendix

Tables 3 and 4.

**Table 3:** Inflection points in the temporal trends of morbidity and mortality indicators for covid-19 among people aged 60 years or older. Brazil, 2020–22

Indicators	Joinpoint	EW/Year		Duration in weeks	WPC	95% IC		*P*-Value
		Start	The end			Low	High	
Hospitalization	0	8/2020	15/2020	7	143.8	124.8	164.3	< 0.001
	1	15/2020	43/2020	28	-2.0	-3.0	-0.9	< 0.001
	2	43/2020	11/2021	21	6.3	4.5	8.0	< 0.001
	3	11/2021	51/2021	40	-7.6	-8.1	-7.0	< 0.001
	4	51/2021	3/2022	4	110.2	55.3	184.7	< 0.001
	5	3/2022	13/2022	10	-35.5	-38.5	-32.4	< 0.001
Severity^a^	0	8/2020	10/2020	2	31.1	16.5	47.6	< 0.001
	1	10/2020	17/2020	7	-6.8	-8.6	-4.9	< 0.001
	2	17/2020	34/2020	17	1.0	0.5	1.4	< 0.001
	3	34/2020	20/2021	39	-0.2	-0.3	-0.1	0.001
	4	20/2021	43/2021	23	0.9	0.6	1.2	< 0.001
	5	43/2021	13/2022	22	-1.6	-1.8	-1.3	< 0.001
Case fatality rate	0	8/2020	16/2020	8	3.9	2.4	5.4	< 0.001
	1	16/2020	43/2020	27	-1.4	-1.7	-1.2	< 0.001
	2	43/2020	10/2021	20	1.4	1.0	1.8	< 0.001
	3	10/2021	39/2021	29	-0.9	-1.1	-0.7	< 0.001
	4	39/2021	9/2022	22	0.1	-0.3	0.4	0.655
	5	9/2022	13/2022	4	-6.9	-10.7	-3.0	0.001
Mortality	0	8/2020	11/2020	3	30.7	-20.4	114.6	0.286
	1	11/2020	14/2020	3	1271.0	408.7	3595.1	< 0.001
	2	14/2020	18/2021	57	1.9	1.3	2.5	< 0.001
	3	18/2021	52/2021	34	-8.1	-9.3	-6.9	< 0.001
	4	52/2021	5/2022	5	71.3	25.2	134.4	0.001
	5	5/2022	13/2022	8	-29.9	-37.1	-21.9	< 0.001

^a^Covid-19 cases admitted to the ICU and/or who received invasive ventilatory support

**Table 4:** Inflection points in the temporal trends of morbidity and mortality indicators for covid-19 among people aged 18 to 59 years. Brazil, 2020–22

Indicators	Joinpoint	EW/Year		Duration in weeks	WPC	95% IC		*P*-Value
		Start	The end			Low	High	
Hospitalization	0	8/2020	15/2020	7	145.0	124.5	167.4	< 0.001
	1	15/2020	42/2020	27	-3.5	-4.6	-2.3	< 0.001
	2	42/2020	19/2021	30	6.7	5.6	7.8	< 0.001
	3	19/2021	49/2021	30	-13.3	-14.2	-12.4	< 0.001
	4	49/2021	3/2022	6	57.8	36.3	82.7	< 0.001
	5	3/2022	13/2022	10	-33.7	-37.0	-30.2	< 0.001
Severity^a^	0	8/2020	11/2020	3	17.3	7.3	28.3	0.001
	1	11/2020	15/2020	4	-10.4	-18.0	-1.9	0.017
	2	15/2020	49/2021	87	0.4	0.4	0.5	< 0.001
	3	49/2021	2/2022	5	-5.3	-10.5	0.2	0.060
	4	2/2022	13/2022	11	1.9	0.7	3.1	0.003
Case fatality rate	0	8/2020	17/2020	9	3.7	0.4	7.1	0.028
	1	17/2020	44/2020	27	-1.7	-2.3	-1.0	< 0.001
	2	44/2020	13/2021	22	3.5	2.5	4.4	< 0.001
	3	13/2021	30/2021	17	-3.2	-4.5	-1.9	< 0.001
	4	20/2021	13/2022	35	0.7	0.2	1.1	0.003
Mortality	0	8/2020	17/2020	9	183.4	161.2	207.6	< 0.001
	1	17/2020	46/2020	29	-4.3	-5.7	-2.9	< 0.001
	2	46/2020	20/2021	27	9.9	8.1	11.7	< 0.001
	3	20/2021	51/2021	31	-13.4	-14.6	-12.3	< 0.001
	4	51/2021	5/2022	6	52.5	24.8	86.3	< 0.001
	5	5/2022	13/2022	8	-27.6	-34.4	-20.2	< 0.001

^a^Covid-19 cases admitted to the ICU and/or who received invasive ventilatory support

## Abbreviations

BIC3: Bayesian Information Traditional Criteria
Covid-19: Coronavirus Disease 2019
E-SUS APS: Primary Health Care Information System
IBGE: Brazilian Institute of Geography and Statistics
95% CI: 95% Confidence Interval
IRR: Incidence-Rate Ratios
USA: United States of America
WHO: World Health Organization
RNDS: National Health Data Network
SAGE: Strategic Advisory Group of Experts on Immunization
SARS-CoV-2: Severe Acute Respiratory Syndrome Coronavirus 2
EW: Epidemiological Week
SI-PNI: National Immunization Program Information System
SIVEP-Gripe: Influenza Epidemiological Surveillance Information System
SARS: Severe Acute Respiratory Syndrome
SUS: Unified Health System
ICU: Intensive Care Unit
VOCs: Variants of Concern
AWPC: Average Weekly Percentage Change
WPC: Weekly Percentage Change
